# Supplementary material for: Untargeted LC-MS/MS Metabolomics Study of HO-AAVPA and VPA on Breast Cancer Cell Lines
Source: Int J Mol Sci. 2023 Sep 26;24(19):14543. doi: 10.3390/ijms241914543 (PMC10572250; doi:10.3390/ijms241914543)
Supplement: Supplementary file 1 [file ijms-24-14543-s001.zip › ijms-2534689-supplementary.pdf]

Untargeted LC–MS/MS Metabolomics Study of HO-AAVPA and VPA on Breast Cancer Cell Lines

Alan Rubén Estrada-Pérez [1], Juan Benjamín García-Vázquez [1], Humberto L. Mendoza-Figueroa [1], Martha Cecilia Rosales-Hernández [2], Cynthia Fernández-Pomares [1] and José Correa-Basurto [1,*]

[1] Laboratorio de Diseño y Desarrollo de Nuevos Fármacos e Innovación Biotecnológica, Escuela Superior de Medicina, Instituto Politécnico Nacional, Plan de San Luis y Díaz Mirón s/n, Casco de Santo Tomás, Ciudad de México 11340, Mexico

[2] Laboratorio de Biofísica y Biocatálisis, Sección de Estudios de Posgrado e Investigación, Escuela Superior de Medicina, Instituto Politécnico Nacional, Plan de San Luis y Díaz Mirón s/n, Casco de Santo Tomás, Ciudad de México 11340, Mexico

* Correspondence: jcorreab@ipn.mx

Supplementary Materials

Table S1. Annotation values of putative metabolites dysregulated in MDA-MB-231 cells.

Metabolite	RT (min)	Mass	HMDB ID	Score MS	Fit	RFit	Purity	Method
Aspidospermatine	1.609	384.2045	HMDB0030358	82.87	0.92	0.97	0.95	RP-ESI-Negative
Arachidonic acid	4.228	304.2409	HMDB0001043	99.35	0.95	0.96	0.92	RP-ESI-Negative
PA(16:0/16:0)	13.62	694.4778	HMDB0000674	99.74	0.85	0.83	0.8	RP-ESI-Negative
PE(14:0/22:5(4Z,7Z,10Z,13Z,16Z))	16.991	737.4987	HMDB0008845	98.84	0.86	0.9	0.84	RP-ESI-Negative
PE(20:0/14:0)	17.234	765.5505	HMDB0009217	99.69	0.95	0.98	0.97	RP-ESI-Negative
Sphinganine	2.04	301.2976	HMDB0000269	85.08	0.98	0.88	0.91	RP-ESI-Positive
4,5-Dihydro-1-benzoxepin-3(2H)-one	2.339	162.068	HMDB0029714	73.11	0.97	0.82	0.86	RP-ESI-Positive
N-trans-Feruloyloctopamine	3.984	351.0971	HMDB0032805	72.37	0.98	0.8	0.88	RP-ESI-Positive
PC(18:4(6Z,9Z,12Z,15Z)/14:0)	7.677	763.4506	HMDB0008229	74.85	0.96	0.97	0.97	RP-ESI-Positive
PE(15:0/14:0)	16.67	649.4721	HMDB0008887	99.02	0.97	1	0.99	RP-ESI-Positive
PE(14:0/15:0)	16.998	649.468	HMDB0008823	99.66	0.89	0.91	0.83	RP-ESI-Positive
PS(18:0/18:0)	19.188	791.5718	HMDB0012378	78.69	1	0.89	0.97	RP-ESI-Positive
PC(14:0/14:0)	19.417	677.4936	HMDB0007866	98.64	0.99	0.99	0.99	RP-ESI-Positive
TG(15:0/22:6(4Z,7Z,10Z,13Z,16Z,19Z)/22:6 (4Z,7Z,10Z,13Z,16Z,19Z))	30.187	974.6757	HMDB0043799	97.97	0.99	0.9	0.99	RP-ESI-Positive
TG(15:0/20:4(5Z,8Z,11Z,14Z)/22:6 (4Z,7Z,10Z,13Z,16Z,19Z))	30.456	950.6765	HMDB0043538	97.42	0.94	0.89	0.9	RP-ESI-Positive
TG(14:0/22:4(7Z,10Z,13Z,16Z)/14:0)	31.209	864.6598	HMDB0042691	98.22	0.9	0.92	0.88	RP-ESI-Positive
Cer(d18:0/26:1(17Z))	32.369	715.6254	HMDB0011772	80.35	0.98	0.96	0.92	RP-ESI-Positive
Cer(d18:0/26:0)	33.189	717.6418	HMDB0011771	84.16	0.94	0.88	0.89	RP-ESI-Positive
TG(20:2n6/18:0/20:2n6)	33.24	948.7535	HMDB0053303	96.18	0.93	0.89	0.86	RP-ESI-Positive

Table S2. Annotation values of putative metabolites dysregulated in MCF7.

Metabolite	RT (min)	Mass	HMDB ID	Score MS	Fit	RFit	Purity	Method
Phenol sulphate	1.385	173.9983	HMDB0060015	72.91	0.84	0.87	0.81	HILIC-ESI-Negative
2-Methyl-3-ketovaleric acid	1.653	130.0629	HMDB0000408	87.41	0.94	0.97	0.92	HILIC-ESI-Negative
Palmitoyl glucuronide	1.934	418.2914	HMDB0010331	92.13	0.94	0.99	0.8	HILIC-ESI-Negative
Pyrogallol-2-O-sulphate	1.938	205.9884	HMDB0060018	97.51	0.88	0.84	0.88	HILIC-ESI-Negative
Valdiate	1.938	310.1783	HMDB0040980	85.38	0.92	0.86	0.87	HILIC-ESI-Negative
2,6-Di-tert-butyl-1,4-benzenediol	1.94	222.1612	HMDB0040178	84.85	0.91	0.97	0.84	HILIC-ESI-Negative
1-Hydroxy-2-pentanone	1.959	102.0681	HMDB0059678	87.57	0.99	0.98	0.98	HILIC-ESI-Negative
Uracil	2.006	112.0272	HMDB0000300	87.83	1	0.92	0.9	HILIC-ESI-Negative
3'-Hydroxy-3,4,5,4'-tetramethoxystilbene	2.058	302.1142	HMDB0041653	78.21	0.85	0.87	0.81	HILIC-ESI-Negative
Acetoxyacetone	2.173	116.0471	HMDB0034466	87.8	0.94	0.96	0.92	HILIC-ESI-Negative
Deoxyfructosazine	2.322	418.1186	HMDB0038696	80.38	0.94	0.99	0.82	HILIC-ESI-Negative
Portulacaxanthin II	2.322	420.1159	HMDB0012281	87.37	0.89	0.84	0.88	HILIC-ESI-Negative
Leucyl-Leucine	2.638	244.1783	HMDB0028933	99.7	0.99	1	0.82	HILIC-ESI-Negative
Homocysteinesulfinic acid	3.361	167.0253	HMDB0006462	99.25	0.86	0.91	0.92	HILIC-ESI-Negative
N-Acetyl-L-methionine	3.381	191.0612	HMDB0011745	77.58	0.94	0.95	0.91	HILIC-ESI-Negative
Pantothenic acid	3.86	219.1106	HMDB0000210	86.81	1	0.95	0.92	HILIC-ESI-Negative
Pantothenic acid	3.86	219.1106	HMDB0000210	86.81	1	0.95	0.92	HILIC-ESI-Negative
Phenylalanyl-Glycine	4.214	222.1001	HMDB0028995	86.32	0.97	0.82	0.86	HILIC-ESI-Negative
Sakacin P	4.564	216.1105	HMDB0038239	86.37	0.98	0.83	0.89	HILIC-ESI-Negative
(±)-2,2'-Iminobispropanoic acid	4.598	161.0688	HMDB0033747	98.97	0.96	0.99	0.97	HILIC-ESI-Negative
Gamma-glutamyl-Phenylalanine	5.061	293.1373	HMDB0029156	85.22	1	0.97	0.91	HILIC-ESI-Negative
Racemethionine	5.566	149.0507	HMDB0033951	79.02	0.88	0.9	0.89	HILIC-ESI-Negative

L-Aspartate-semialdehyde	5.986	117.0423	HMDB0012249	87.25	0.85	0.81	0.8	HILIC-ESI-Negative
Phenylalanylglutamine	5.987	293.1372	HMDB0028993	85.89	1	1	0.97	HILIC-ESI-Negative
L-N-(3-Carboxypropyl)glutamine	6.098	232.1054	HMDB0029393	85.87	0.92	0.95	0.92	HILIC-ESI-Negative
Valyl-Alanine	6.202	188.1159	HMDB0029120	87.37	0.93	0.84	0.85	HILIC-ESI-Negative
Alanyl-Glycine	6.572	146.0686	HMDB0006899	79.78	0.82	0.83	0.82	HILIC-ESI-Negative
Glycyl-Hydroxyproline	6.599	188.0793	HMDB0011173	86.71	0.97	0.96	0.9	HILIC-ESI-Negative
Ethyl nitrite	7.112	75.0319	HMDB0031239	87.75	0.9	0.9	0.84	HILIC-ESI-Negative
Methoxyacetic acid	9.013	90.0315	HMDB0041929	86.2	0.85	0.87	0.8	HILIC-ESI-Negative
L-Asparagine	10.002	132.0533	HMDB0000168	83.89	0.88	0.9	0.82	HILIC-ESI-Negative
Dihydroandrosterone	2.304	210.1008	HMDB0015441	84.6	1	0.95	0.9	HILIC-ESI-Positive
Kanzonol O	2.887	330.1981	HMDB0000554	84.37	0.97	0.82	0.86	HILIC-ESI-Positive
LysoPC(16:1(9Z))	2.975	382.1421	HMDB0041102	85.61	0.98	0.8	0.88	HILIC-ESI-Positive
1-Nitroheptane	3.457	493.3159	HMDB0010383	79.2	0.96	0.97	0.97	HILIC-ESI-Positive
Styrene	3.503	145.1102	HMDB0013811	98.78	0.94	0.94	0.91	HILIC-ESI-Positive
(S)-Homostachydrine	3.549	157.1102	HMDB0033433	86.44	0.85	0.82	0.86	HILIC-ESI-Positive
Buprenorphine	3.684	467.3006	HMDB0015057	99.43	0.95	0.99	0.99	HILIC-ESI-Positive
7-Methylguanosine	4.31	297.1072	HMDB0001107	85.29	0.92	0.91	0.92	HILIC-ESI-Positive
cis-4-Decenedioic acid	4.381	200.1052	HMDB0000603	87.19	0.99	0.9	0.99	HILIC-ESI-Positive
L-Hexanoylcarnitine	4.381	259.1788	HMDB0000756	99.2	0.87	0.8	0.82	HILIC-ESI-Positive
5-Aminoimidazole-4-carboxamide	4.915	126.0543	HMDB0003192	86.42	0.94	0.91	0.9	HILIC-ESI-Positive
5-Butyltetrahydro-2-oxo-3-furancarboxylic acid	5.728	186.0898	HMDB0030992	85.87	0.9	0.92	0.88	HILIC-ESI-Positive
4-Hydroxy-2-butenoic acid gamma-lactone	9.052	84.0212	HMDB0032330	87.92	0.98	0.95	0.92	HILIC-ESI-Positive
Leucyl-Proline	9.514	228.1472	HMDB0011175	86.55	0.93	0.91	0.9	HILIC-ESI-Positive
Pyro-L-glutaminyl-L-glutamine	9.76	257.101	HMDB0039229	84.54	0.94	0.88	0.92	HILIC-ESI-Positive
3'-O-Methyladenosine	9.988	281.1122	HMDB0006023	99.56	0.93	0.89	0.86	HILIC-ESI-Positive
N-Ethylglycine	10.306	103.063	HMDB0041945	87.23	0.99	0.91	0.94	HILIC-ESI-Positive
N-Acetylglutamine	13.357	188.0799	HMDB0006029	87.16	0.89	0.84	0.9	HILIC-ESI-Positive
3-Acetamidobutanal	15.119	129.079	HMDB0059649	87.84	0.99	1	0.92	HILIC-ESI-Positive
LysoPE(0:0/18:0)	3.47	481.3172	HMDB0011129	96.91	0.89	0.92	0.84	RP-ESI-Negative
LysoPE(0:0/16:0)	3.613	453.2859	HMDB0011473	98.04	0.85	0.86	0.81	RP-ESI-Negative
LysoPE(18:1(9Z)/0:0)	3.902	479.3022	HMDB0011506	98.17	0.97	0.92	0.8	RP-ESI-Negative
LysoPE(18:0/0:0)	5.303	481.3172	HMDB0011130	99.29	0.94	0.96	0.9	RP-ESI-Negative
PS(18:1(9Z)/16:0)	13.294	761.5202	HMDB0012387	99.07	0.99	0.99	0.94	RP-ESI-Negative
Cer(d18:1/14:0)	18.931	509.4802	HMDB0011773	98.52	0.94	0.94	0.93	RP-ESI-Negative
PE(18:1(9Z)/14:0)	18.944	689.4994	HMDB0009052	95.22	0.88	0.9	0.87	RP-ESI-Negative
PE(14:1(9Z)/20:1(11Z))	19.288	715.516	HMDB0008867	95.67	0.85	0.82	0.83	RP-ESI-Negative
PE(14:1(9Z)/22:2(13Z,16Z))	19.848	741.5309	HMDB0008876	97.35	0.95	0.98	0.86	RP-ESI-Negative

Cer(d18:0/14:0)	19.935	511.4958	HMDB0011759	98.9	0.94	0.92	0.94	RP-ESI-Negative
PE(22:2(13Z,16Z)/14:1(9Z))	20.906	741.5303	HMDB0009548	98.94	0.99	0.92	0.92	RP-ESI-Negative
PE(16:1(9Z)/24:1(15Z))	20.975	791.5476	HMDB0009684	95.47	0.98	0.97	0.91	RP-ESI-Negative
PE(14:0/22:2(13Z,16Z))	21.66	743.5479	HMDB0008843	97.1	0.81	0.81	0.82	RP-ESI-Negative
PE(24:0/14:0)	21.933	743.5464	HMDB0009547	97.27	0.97	0.96	0.95	RP-ESI-Negative
Cer(d18:0/16:0)	22.429	539.5272	HMDB0011760	98.3	0.94	0.96	0.9	RP-ESI-Negative
PE-NMe(18:1(9Z)/18:1(9Z))	22.655	757.5617	HMDB0010565	99.4	0.99	0.99	0.94	RP-ESI-Negative
'PE(22:2(13Z,16Z)/16:1(9Z))'	23.171	769.5616	HMDB0009551	99.54	0.97	0.82	0.86	RP-ESI-Negative
PE(18:1(11Z)/24:1(15Z))	23.302	873.6453	HMDB0009047	98.75	0.98	0.8	0.88	RP-ESI-Negative
2-O-(4,7,10,13,16,19-Docosahexaenoyl)-1-O-hexadecylglycero-3-phosphocholine	23.309	851.6024	HMDB0013409	75.72	0.96	0.97	0.97	RP-ESI-Negative
PE(16:0/22:2(13Z,16Z))	23.693	771.578	HMDB0008942	96.98	0.96	0.97	0.97	RP-ESI-Negative
1-[1,4-Dihydro-4-nonyl-5-(1-oxodecyl)-3-pyridinyl]-1-dodecanone	23.851	603.5186	HMDB0035518	75.05	0.97	0.89	0.96	RP-ESI-Negative
PE(24:1(15Z)/14:0)	25.536	773.5927	HMDB0009745	98.41	0.89	0.91	0.83	RP-ESI-Negative
PE(24:1(15Z)/16:1(9Z))	25.552	799.6087	HMDB0009749	99.04	0.93	0.89	0.97	RP-ESI-Negative
1,1'-(1,4-Dihydro-4-nonyl-3,5-pyridinediyl)bis[1-decanone]	25.816	631.5505	HMDB0035519	71.09	0.99	0.99	0.99	RP-ESI-Negative
1,1'-(1,4-Dihydro-4-nonyl-3,5-pyridinediyl)bis[1-dodecanone]	25.816	631.5505	HMDB0035519	71.09	0.99	0.99	0.99	RP-ESI-Negative
Campesteryl linoleate	26.13	708.6012	HMDB0036287	78.97	0.92	0.91	0.92	RP-ESI-Negative
PE-NMe2(16:0/18:1(9Z))	26.134	745.5652	HMDB0010568	70.04	0.95	0.95	0.86	RP-ESI-Negative
PE(24:1(15Z)/18:1(9Z))	26.963	827.6396	HMDB0009752	98.58	0.9	0.94	0.92	RP-ESI-Negative
Cer(d18:0/24:1(15Z))	27.711	649.636	HMDB0011769	96.78	1	0.99	1	RP-ESI-Negative
PE(24:1(15Z)/18:0)	28.133	829.6556	HMDB0009750	99.17	0.99	0.96	0.97	RP-ESI-Negative
CL(18:2(9Z,12Z)/18:0/18:2(9Z,12Z)/16:1(9Z))	28.672	677.668	HMDB0011772	96.91	0.95	0.99	0.97	RP-ESI-Negative
Aprobarbital	29.168	1455.0164	HMDB0058719	90.01	0.84	0.82	0.87	RP-ESI-Negative
Acetophenazine	1.721	433.1782	HMDB0015196	70.99	0.83	70.99	0.83	RP-ESI-Positive
LysoPC(14:0)	2.553	467.3009	HMDB0010379	99.48	0.94	0.93	0.84	RP-ESI-Positive
LysoPC(16:0)	3.775	495.3327	HMDB0010382	99.27	0.96	0.88	0.88	RP-ESI-Positive
PC(14:0/14:0)	15.438	677.4999	HMDB0007866	99.24	0.96	0.97	0.98	RP-ESI-Positive
PC(18:2(9Z,12Z)/18:4(6Z,9Z,12Z,15Z))	15.441	777.531	HMDB0008142	99.49	0.95	0.98	0.84	RP-ESI-Positive
CL(18:1(9Z)/16:0/18:1(9Z)/18:0)	16.28	1433.0405	HMDB0058322	72.85	0.85	0.86	0.81	RP-ESI-Positive
PE(22:6(4Z,7Z,10Z,13Z,16Z,19Z)/16:1(9Z))	16.429	761.4997	HMDB0009683	99.14	0.97	0.92	0.8	RP-ESI-Positive
PC(18:2(9Z,12Z)/18:3(6Z,9Z,12Z))	16.562	779.546	HMDB0008140	99.61	0.95	0.9	0.93	RP-ESI-Positive
PC(18:3(6Z,9Z,12Z)/16:0)	17.024	755.5464	HMDB0008166	99.46	0.98	0.97	0.98	RP-ESI-Positive
PE(14:0/22:5(4Z,7Z,10Z,13Z,16Z))	17.109	737.4995	HMDB0008845	92.48	0.89	0.91	0.83	RP-ESI-Positive
PE(22:5(7Z,10Z,13Z,16Z,19Z)/14:0)	17.606	737.4993	HMDB0009646	83.55	0.93	0.89	0.97	RP-ESI-Positive
PE(20:3(8Z,11Z,14Z)/14:0)	17.893	713.4996	HMDB0009349	99.81	0.99	0.99	0.99	RP-ESI-Positive
Cohibin D	18.705	576.5114	HMDB0035398	99.4	0.9	0.92	0.81	RP-ESI-Positive
PC(16:0/15:0)	18.979	719.5465	HMDB0007967	99.54	1	0.99	1	RP-ESI-Positive
PC(14:1(9Z)/15:0)	19.111	689.4998	HMDB0007901	99.82	0.95	1	0.97	RP-ESI-Positive
CL(18:1(9Z)/18:0/18:2(9Z,12Z)/18:0)	19.377	1473.074	HMDB0058362	75.81	0.89	0.92	0.84	RP-ESI-Positive
PE(20:2(11Z,14Z)/14:0)	19.454	715.5151	HMDB0009283	99.82	0.85	1	0.81	RP-ESI-Positive
PC(18:3(6Z,9Z,12Z)/18:0)	19.5	783.5777	HMDB0008168	99.75	0.97	0.92	0.8	RP-ESI-Positive
PC(18:3(6Z,9Z,12Z)/15:0)	20.02	741.5313	HMDB0008165	99.68	0.94	0.96	0.91	RP-ESI-Positive

PC(20:5(5Z,8Z,11Z,14Z,17Z)/15:0)	20.21	765.531	HMDB0008494	85.29	1	0.99	0.94	RP-ESI-Positive
PC(15:0/18:3(9Z,12Z,15Z))	20.346	741.5309	HMDB0007942	99.77	0.98	0.95	0.9	RP-ESI-Positive
PC(18:1(9Z)/18:1(9Z))	21.214	785.5934	HMDB0000593	99.66	0.92	0.86	0.9	RP-ESI-Positive
PC(16:1(9Z)/15:0)	21.534	717.5307	HMDB0008000	99.86	0.94	0.94	0.93	RP-ESI-Positive
Ceramide (d18:1/16:0)	21.649	537.5118	HMDB0004949	99.76	0.89	0.89	0.8	RP-ESI-Positive
PC(18:2(9Z,12Z)/15:0)	21.827	743.5465	HMDB0008132	99.79	0.91	0.95	0.83	RP-ESI-Positive
PC(18:2(9Z,12Z)/20:1(11Z))	21.886	811.6086	HMDB0008144	99.78	0.92	1	0.87	RP-ESI-Positive
PC(22:5(7Z,10Z,13Z,16Z,19Z)/20:0)	22.913	863.6398	HMDB0008701	99.49	0.99	0.94	0.93	RP-ESI-Positive
PE(24:1(15Z)/18:4(6Z,9Z,12Z,15Z))	23.157	821.5933	HMDB0009756	91.03	0.85	0.88	0.81	RP-ESI-Positive
PC(22:5(7Z,10Z,13Z,16Z,19Z)/22:1(13Z))	23.26	889.6549	HMDB0008710	99.25	1	0.96	0.92	RP-ESI-Positive
PC(18:2(9Z,12Z)/20:0)	23.571	821.5939	HMDB0009531	92.27	0.99	0.98	0.82	RP-ESI-Positive
SM(d18:0/26:1(17Z))	23.578	813.6248	HMDB0008143	99.82	1	1	0.99	RP-ESI-Positive
Cer(d18:1/24:1(15Z))	27.4	647.6215	HMDB0004953	95.55	0.93	0.89	0.97	RP-ESI-Positive
Cer(d18:1/24:0)	27.857	649.6367	HMDB0004956	99.6	0.99	0.99	0.99	RP-ESI-Positive
Glucosylceramide (d18:1/26:0)	28.113	839.7203	HMDB0004977	94.33	0.92	0.91	0.92	RP-ESI-Positive
PE(18:0/24:1(15Z))	28.217	829.6555	HMDB0009014	99.78	0.87	0.8	0.82	RP-ESI-Positive
Cer(d18:1/26:0)	29.335	677.6682	HMDB0004955	99.74	0.99	0.9	0.99	RP-ESI-Positive
TG(18:4(6Z,9Z,12Z,15Z)/16:0/18:4(6Z,9Z,12Z,15Z))	29.37	884.6291	HMDB0055369	88.08	0.94	0.89	0.9	RP-ESI-Positive
TG(14:1(9Z)/16:0/14:1(9Z))	29.796	768.6237	HMDB0047770	99.35	0.9	0.92	0.88	RP-ESI-Positive
CL(18:1(9Z)/16:1(9Z)/18:1(9Z)/16:1(9Z))	29.821	1400.971	HMDB0058384	96.27	0.98	0.95	0.92	RP-ESI-Positive
TG(14:0/14:0/20:4(5Z,8Z,11Z,14Z))	29.888	836.6292	HMDB0042080	90.57	0.93	0.91	0.9	RP-ESI-Positive
TG(14:1(9Z)/15:0/16:1(9Z))	30.022	798.6143	HMDB0047748	88.81	0.94	0.88	0.89	RP-ESI-Positive
TG(16:1(9Z)/14:0/18:3(9Z,12Z,15Z))	30.171	836.6291	HMDB0048428	99.23	0.93	0.89	0.86	RP-ESI-Positive
TG(20:3n6/14:0/18:3(9Z,12Z,15Z))	30.22	872.6947	HMDB0053658	90.81	0.92	0.97	0.95	RP-ESI-Positive
Ubisemiquinone	30.324	862.684	HMDB0013111	99.69	0.95	0.96	0.92	RP-ESI-Positive
TG(16:1(9Z)/14:1(9Z)/18:1(9Z))	30.378	822.6706	HMDB0048569	99.76	0.85	0.83	0.8	RP-ESI-Positive
TG(14:0/22:4(7Z,10Z,13Z,16Z)/14:0)	30.771	848.6856	HMDB0042691	99.14	0.95	0.98	0.97	RP-ESI-Positive
SM(d18:1/26:0)	30.773	843.7297	HMDB0011698	98.43	0.86	0.81	0.8	RP-ESI-Positive
